# Miscellaneous Items

**Published:** 1856-04

**Authors:** 


### Miscellaneous Items.

*A Spiritual Doctor,*—A correspondent of the *American Medical Gazette* says of one of these humbugs in St. Louis, that he is attracting more notice, and reaping more profits from his vocation, than any two practitioners in the city.

"Like some of the ancient apostles (though not a fisherman,) he is of humble origin—having been a *boatman;* and while at a game of cards, on the river, was suddenly 'impressed' that he had a mission to perform different from that in which he was engaged. He hastened home by railroad speed—found several of his family very sick,—dismissed the physician in attendance, and by his own magic wand restored them to health. Thus commenced his career. He is sought after by all classes. The fashionable and wealthy dance attendance at his office; and often may be seen in front of his door half a dozen or more carriages waiting their turn. He claims supernatural powers and very wisely predicts that this extraordinary power will not continue with him very long." Alas for poor human nature.

*A Man with Eleven Wives.*—There is now living in the neighborhood of Glasgow, a lady in her 107th year; and very recently another female is reported to have died at the age of 101; whilst a carpenter, named John Walney, died in Glasgow during 1757, actually 124 years old. This man is stated to have married eleven wives, all of whom he buried; and of his seventeen children five survived him, whose ages amounted to 326 years collectively.

*Dr. Beale's Reception.*—A reception has recently been given, by upwards of fifty members of the dental profession in New York, to Dr. Beale and his family, at the house of Dr. Brown, near Jones street, to congratulate him, after the act of liberation awarded to him by the Governor of Pennsylvania.

*Consumption Hospital in New York.*—The trustees of this institution have made an appeal for funds to aid them in erecting a suitable building. This appeal is headed by Peter Cooper. Drs. Alonzo Clark and John H. Griscom are among the medical names. We can hardly conceive of a more useful, a more indispensable charitable institution, in a great capital like New York, where from one-fourth to one-seventh of all the deaths are from phthisis. We sincerely hope the trustees may be successful in obtaining ample means for constructing a hospital suitable to the wants of New York.

